# Model-based Assessment of the Effect of Contact Precautions Applied to Surveillance-detected Carriers of Carbapenemase-producing Enterobacteriaceae in Long-term Acute Care Hospitals

**DOI:** 10.1093/cid/ciz557

**Published:** 2019-09-13

**Authors:** Damon J A Toth, Karim Khader, Alexander Beams, Matthew H Samore

**Affiliations:** 1 Department of Veterans Affairs Salt Lake City Health Care System, Salt Lake City; 2 Department of Internal Medicine (Epidemiology), University of Utah, Salt Lake City; 3 Department of Mathematics, University of Utah, Salt Lake City

**Keywords:** contact precautions, carbapenem-resistant Enterobacteriaceae, transmission, active surveillance, mathematical model

## Abstract

**Background:**

An intervention that successfully reduced colonization and infection with carbapenemase-producing Enterobacteriaceae (CPE) in Chicago-area long-term acute-care hospitals included active surveillance and contact precautions. However, the specific effects of contact precautions applied to surveillance-detected carriers on patient-to-patient transmission are unknown, as other, concurrent intervention components or changes in facility patient dynamics also could have affected the observed outcomes.

**Methods:**

Using previously published data from before and after the CPE intervention, we designed a mathematical model with an explicit representation of postintervention surveillance. We estimated preintervention to postintervention changes of 3 parameters: β, the baseline transmission rate excluding contact precaution effects; $\delta_{b}$, the rate of a CPE carrier progressing to bacteremia; and $\delta_{c}$, the progression rate to nonbacteremia clinical detection.

**Results:**

Assuming that CPE carriers under contact precautions transmit carriage to other patients at half the rate of undetected carriers, the model produced no convincing evidence for a postintervention change in the baseline transmission rate β (+2.1% [95% confidence interval {CI}, −18% to +28%]). The model did find evidence of a postintervention decrease for $\delta_{b}$ (−41% [95% CI, −60% to −18%]), but not for $\delta_{c}$ (−7% [95% CI, −28% to +19%]).

**Conclusions:**

Our results suggest that contact precautions for surveillance-detected CPE carriers could potentially explain the observed decrease in colonization by itself, even under conservative assumptions for the effectiveness of those precautions for reducing cross-transmission. Other intervention components such as daily chlorhexidine gluconate bathing of all patients and hand-hygiene education and adherence monitoring may have contributed primarily to reducing rates of colonized patients progressing to bacteremia.

## Teaser

We mathematically modeled a bundled intervention that successfully reduced carbapenemase-producing Enterobacteriaceae in long-term acute care hospitals. Results show that contact precautions for surveillance-detected carriers plausibly can explain the observed reduction in carriage prevalence without substantial contribution from other intervention components.

Infections caused by multidrug-resistant organisms have limited available treatments and pose a significant health threat [1]. Some drug-resistant organisms have shown the ability to disseminate rapidly among patients within and between certain healthcare facilities [2], suggesting that focused and/or coordinated efforts to reduce patient-to-patient transmission could provide substantial population benefit if implemented efficiently [3].

Interventions to interrupt the transmission of organisms among patients in healthcare facilities often take a “bundled” approach, comprising multifaceted efforts that may include, for example, instituting active surveillance for asymptomatic carriers; contact precautions such as glove and gown use, isolation, and/or cohorting for identified carriers; healthcare worker education/monitoring for hygiene behaviors; decontamination procedures applied to patients and/or environmental surfaces; and antibiotic stewardship [4, 5]. Applying a bundled approach can benefit the target facilities by maximizing the potential for successfully reducing infections, but the interpretation of the outcomes is difficult, particularly when attempting to assess the role that particular components of the bundle may have played in a successful reduction [6].

Active surveillance combined with contact precautions applied to detected carriers is an intervention component of high interest due to its high cost and the difficulty in directly assessing its effectiveness. While healthcare facilities instituting new surveillance programs have seen decreases in healthcare-associated infections [4], it has been difficult to convincingly attribute the decreases to contact precautions rather than other concurrent infection control efforts [7]. Also, some facilities have discontinued the use of contact precautions for certain organisms and identified no adverse effects [8], although the interpretation of such data has been questioned [7]. Our approach to contributing insight to these questions is to pair mathematical transmission and infection models with data from before and after bundled interventions, including active surveillance and contact precautions, to help disentangle the potential effects from that of other components.

The specific scenario we model here is an intervention targeted at carbapenemase-producing Enterobacteriaceae (CPE) in a long-term acute care hospital (LTACH) [4]. Due to their composition of acutely ill patients with long length of stay, LTACHs are high-risk settings for infections with CPE and other high-priority organisms [9]. Efforts to interrupt transmission could provide substantial benefit to LTACHs and other facilities linked by patient exchange [10]. Therefore, understanding the role of individual intervention components for reducing transmission could be crucial to achieving maximum efficiency in regional outbreak prevention.

Specifically, we calibrate our model to an intervention that was initiated in Chicago-area LTACHs with nearly 50% prevalence of CPE carriers and significant rates of CPE clinical detection and bacteremia (3.7 and 0.9 per 1000 patient-days, respectively) [4]. After the intervention, CPE carriage prevalence, clinical detection incidence, and bacteremia incidence decreased by approximately 25%, 32%, and 56%, respectively, while importation rates remained stable (Table 1). The facilities did not routinely perform CPE surveillance before the intervention, while the intervention included high rates of surveillance: >90% of patients were rectally cultured for CPE carriage at admission and every other week during their stay, and patients testing positive were placed under contact isolation [4]. However, because other intervention components were implanted concurrently with surveillance and contact precautions, we sought to separate the specific impact of that component using our model.

**Table 1.** Data From Hayden et al Results Applied to Model

Value (Symbol)	Data	(95% CI)
Admission CPE positivity fraction (a)		
Preintervention	0.206	(.191–.223)
Postintervention	0.206	(.191–.223)
Cross-sectional facility CPE positivity fraction (f)		
Preintervention	0.458	(.421–.495)
Postintervention	0.343	(.324–.362)
CPE clinical detection rate per 1000 patient days (d)		
Preintervention	3.7	(3.4–4.0)
Postintervention	2.5	(2.2–2.8)
CPE bacteremia onset rate per 1000 patient-days (b)		
Preintervention	0.9	(.8–1.1)
Postintervention	0.4	(.3–.5)
Inpatient death rate per admission ($p_{d}$)		
Preintervention	0.215	…
Postintervention	0.176	…
Days of stay: mean; 25th, 50th, 75th percentiles (μ, $l_{25}$, $l_{50}$, $l_{75}$)		
Preintervention	33.8; 16, 28, 43	…
Postintervention	30.5; 16, 26, 39	…
Admission surveillance test probability ($s_{a}$)		
Preintervention	0	…
Postintervention	0.911	(.901–.921)
Every other week surveillance test probability ($s_{b}$)		
Preintervention	0	…
Postintervention	0.954	(.948–.960)

Abbreviations: CI, confidence interval; CPE, carbapenemase-producing Enterobacteriaceae.

## METHODS

We developed a mathematical model representing the portion of LTACH inpatients in different states of CPE colonization, infection, and contact precaution status. Each model carries an assumption of a constant patient death rate and a live-discharge hazard that depends on the time since the patient’s admission, using a mixture of gamma distributions (Supplementary Materials). These settings were calibrated to match the statistics reported in the manuscript: in-hospital mortality percentage and mean, median, and interquartile range of hospital stay. Those statistics were reported separately for the preintervention and postintervention periods (Table 1), so we calibrated separate death and length of stay settings for each model.

**Figure 1.**
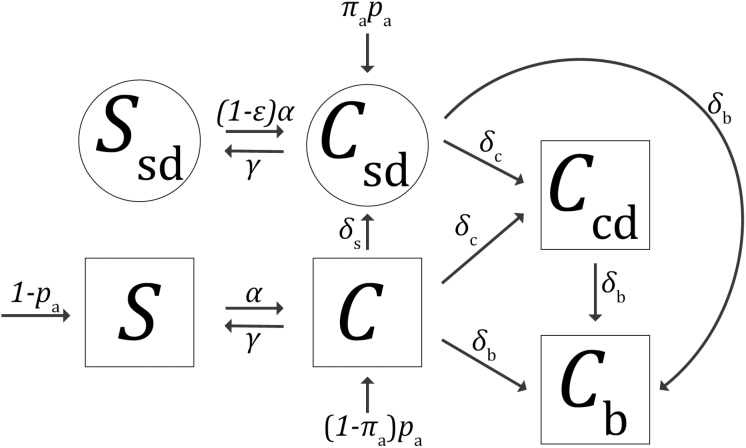
Patient states and state transitions. Squares depict possible states in the preintervention model; circled states are added in the postintervention model. Arrows between shapes depict possible state transitions during the long-term acute care hospital stay and are labeled with rate parameters; arrows into shapes from outside depict possible states at admission and are labeled with probabilities of each admission state. Patients in any state can be removed via death or live discharge (not depicted; see Supplementary Materials).

LTACH inpatients were classified into 6 possible states (Figure 1):

- Susceptible (S): not colonized with CPE; no surveillance detection
- Colonized and undetected (C): undetected CPE carrier
- Colonized and clinically detected ($C_{cd}$): clinically detected CPE carrier; no CPE bacteremia
- Colonized with bacteremia ($C_{b}$): CPE carrier with CPE bacteremia during this stay
- Susceptible and surveillance detected ($S_{sd}$): not colonized with CPE; prior surveillance detection
- Colonized and surveillance detected ($C_{sd}$): surveillance-detected CPE carrier; no CPE clinical detection

In the preintervention scenario, only the first 4 states were possible as there was no surveillance, and postintervention all 6 states were possible. Preintervention, patients were in state S or C at admission, with probability matching the reported admission prevalence data at the start of the intervention, scaled by the assumed test sensitivity. Postintervention, patients could be in the colonized and surveillance-detected state $C_{sd}$ at admission if they tested positive under the admission surveillance component of the intervention. Patients admitted in state C postintervention were either carriers who were not tested, assumed to occur at the reported adherence rate, or carriers who falsely tested negative.

Patients in state S move to state C (acquire colonization due to transmission) at the acquisition rate α proportional to the prevalence of patients in one of the colonized states:

$$\begin{array}{l} \alpha =\beta \left(C+\left(1-\varepsilon \right)\left(C_{sd}+C_{cd}+C_{b}\right)\right) \end{array}$$

The parameter β, the baseline transmission rate, defines how the acquisition rate depends on the prevalence of carriers in the LTACH. A susceptible patient acquires colonization from undetected carriers at rate βC, where C is the prevalence of undetected carriers, and from detected carriers at rate $\beta \left(1-\varepsilon \right)\left(C_{sd}+C_{cd}+C_{b}\right)$, where $C_{sd}$, $C_{cd}$, and $C_{b}$ are the prevalence of carriers in each of the detected states and ε is the effectiveness of contact precautions at reducing transmission compared to the baseline.

Patients in state C could clear colonization at rate γ, progress to bacteremia at rate $\delta_{b}$ or other clinical detection at rate $\delta_{c}$, or, in the postintervention scenario, become surveillance detected (rate $\delta_{s}$) due to the biweekly surveillance component of the intervention, assumed to occur at a rate of once per 14 days and also scaled by the reported adherence and assumed test sensitivity. Surveillance-detected patients in state $C_{sd}$ could also experience bacteremia or other clinical detection, and patients in state $C_{cd}$ (clinically detected but no bacteremia) could also progress to bacteremia. The surveillance-detected susceptible state $S_{sd}$ accounts for uninfected, surveillance-detected carriers who cleared colonization during the stay. Surveillance-positive patients remained under contact precautions for their entire stay during the intervention [4], and for those who may have cleared colonization before discharge, we assumed that those precautions reduced their rate of reacquisition by the same factor, (1−ε), that they reduced transmissibility.

The acquisition rate α, bacteremia progression rate $\delta_{b}$, and progression rate to nonbacteremia clinical detection $\delta_{c}$ were simultaneously calibrated to match the equilibrium cross-sectional carriage prevalence (scaled by the assumed test sensitivity), clinical detection incidence, and bacteremia onset incidence reported in the data, separately for the pre- and postintervention scenarios (Table 1). Then we solved for the baseline transmission rate β that produced the correct acquisition rate under the calibrated model (Table 2 and Supplementary Materials).

**Table 2.** Model Parameters

Parameter	Assumed Value (Range Tested) or Formula
Clearance rate of CPE carriers per day (γ)	1/387 (1/700, 1/50)
Contact precaution effectiveness (ε)	0.5 (0.1–0.9)
Surveillance test sensitivity (σ)	0.85 (0.75–0.95)
CPE importation rate ($p_{a}$)	a/σ
Admission detection probability of CPE importers ($\pi_{a}$)	$s_{a}\sigma$
Within-stay surveillance detection rate per day of CPE carriers ($\delta_{s}$)	$s_{b}\sigma /14$
Death rate/length of stay parameters (ω, $p_{x}$, $\mu_{x}$, $\mu_{g}$, k)	See Supplementary Methods
CPE baseline transmission rate (β)	See Results
CPE bacteremia onset rate of CPE carriers ($\delta_{b}$)	See Results
CPE nonbacteremia clinical detection rate of CPE carriers ($\left(\delta_{c}\right)$	See Results
CPE acquisition rate of susceptible patients (α)	$\beta \left(C+\left(1-\varepsilon \right)\left(C_{sd}+C_{cd}+C_{b}\right)\right)$

Abbreviation: CPE, carbapenemase-producing Enterobacteriaceae.

We compared the postintervention change in β, $\delta_{b}$, and $\delta_{c}$ compared to their preintervention values to draw conclusions about intervention mechanisms under different assumptions for other parameters described below. In particular, because the transmission-reduction effect of placing surveillance-detected carriers under contact precautions was explicitly captured by tallying the patients in state $C_{sd}$ and by the contact precaution effectiveness parameter ε, our estimate for the postintervention change in β represents the intervention’s effect on transmission by mechanisms other than detection and contact precautions.

Estimates for effectiveness of contact precautions ε, the clearance rate for CPE carriage γ, and the surveillance test sensitivity σ were not identifiable from the data [4], so we independently obtained these values from other sources (Table 2). First, we assumed the effectiveness of contact precautions at reducing transmission to be 50%, that is, detected CPE carriers, assumed to be under contact precautions, transmit to other patients at a rate 50% less than undetected carriers. We used this assumption in previous publications [3, 10], based on data from comparing contamination levels on healthcare provider hands after interacting with colonized patients with and without contact precautions [11]. To test the sensitivity of our conclusions to this assumption, we produced alternate results using assumptions of 10%, 30%, 70%, and 90% effectiveness. Second, we assumed a clearance rate of 1/387 per day [12] and tested alternate values of 1/50, 1/200, 1/550, and 1/700 per day. Third, we assumed surveillance test sensitivity of 85% [13, 14] and tested alternate values of 75%, 80%, 90%, and 95%. When varying each parameter, we kept the other 2 at its default value, for a total of 13 parameter combinations.

We also accounted for uncertainty arising from the pre- and postintervention data that were used for parameter values (admission CPE positivity and surveillance adherence fractions), or calibration targets (cross-sectional CPE positivity fraction and clinical detection and bacteremia incidence) in the model. Here, we relied on the reported uncertainty ranges from the source paper [4] (Table 1). Each value with reported uncertainty was derived from a large number of observations [4], justifying the use of a normal distribution approximation to the underlying distribution. We incorporated these assumed parameter distributions into a Latin hypercube sampling scheme [15], which produces random sets of values representative of the multivariate distribution. For each of the 13 fixed combinations of the 3 parameters described in the previous paragraph, we used 1000 combinations of the paper-derived values from the Latin hypercube sampling scheme, to create 95% confidence intervals (CIs) for our results.

## RESULTS

**Figure 2.**
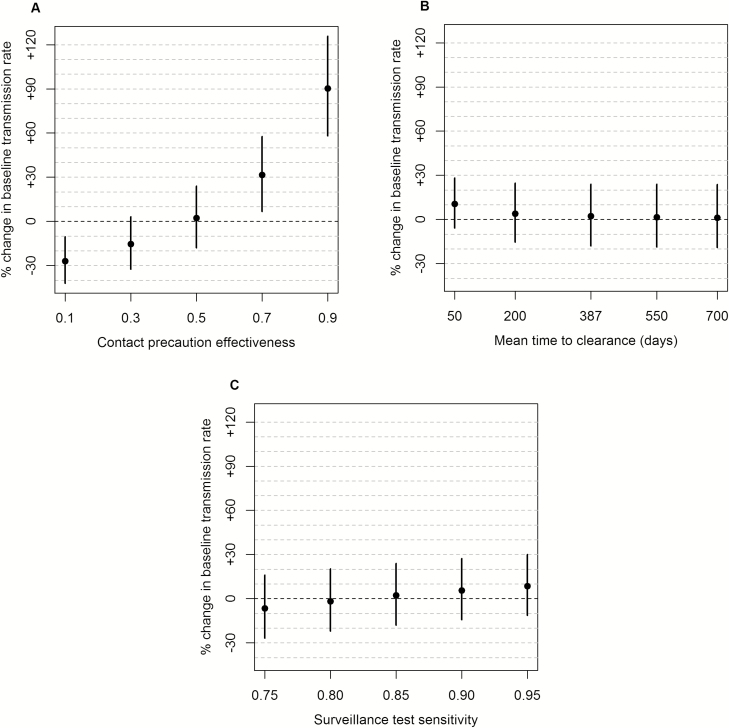
Effect of assumptions on transmission rate results. Vertical axis is the % change from preintervention to postintervention of the result for β, the transmission rate from undetected carbapenemase-producing Enterobacteriaceae (CPE) carriers. Panels show the sensitivity of this result to changes in single parameters from the default (default is the middle value in each panel). Circles: mean results; vertical lines: 95% confidence intervals. *A*, Effectiveness of contact precautions is ε, so the per-capita acquisition rate of nonisolated susceptible patients is $\beta_{pre}\left(C+\left(1-\varepsilon \right)\left(C_{cd}+C_{b}\right)\right)$ preintervention and $\beta_{post}\left(C+\left(1-\varepsilon \right)\left(C_{sd}+C_{cd}+C_{b}\right)\right)$ postintervention. *B*, Mean time to clearance is 1/γ days, where γ is the rate at which non–clinically infected CPE carriers clear colonization and become susceptible to reacquisition. *C*, Surveillance test sensitivity is 1 minus the probability that carbapenemase-producing Enterobacteriaceae carriers falsely test negative at surveillance.

Under our assumed values for the 3 independently based parameters (ε= 0.5, γ= 1/387 per day, σ= 0.85), we estimated the pre- and postintervention values of β, $\delta_{b}$, and $\delta_{c}$ (Table 3). Our estimate for the baseline transmission rate β preintervention was 0.051 per day (95% CI, .044–.060) and postintervention β was 0.052 per day (95% CI, .045–.059). The relative change in β after the intervention was +2.1% (95% CI, −18% to +28%). The latter result was most sensitive to changes in the assumed effect of contact precautions on transmission. Lower assumed effectiveness produced a greater postintervention decrease in β (Figure 2A). With ε= 0.1, preintervention β was 0.047 per day (95% CI, .040–.055), postintervention β was 0.034 per day (95% CI, .029–.040), and the relative change in β was −27% (95% CI, −42% to −10%). With ε= 0.9, preintervention β was 0.056 per day (95% CI, .048–.065), postintervention β was 0.106 per day (95% CI, .096–.117), and the relative change in β was +90% (95% CI, +58% to +126%). Results for the change in β were much less sensitive to the assumed values for the clearance rate and surveillance test sensitivity (Figure 2B and 2C).

**Table 3.** Model Results

Parameter	Estimated Value	(95% CI)
CPE baseline transmission rate (β)		
Preintervention	0.051 per day	(.044–.060)
Postintervention	0.052 per day	(.045–.059)
Relative change after intervention	+2%	(−18% to +28%)
CPE bacteremia onset rate of CPE carriers ($\delta_{b}$)		
Preintervention	0.0018 per day	(.0015–.0021)
Postintervention	0.0010 per day	(.0008–.0014)
Relative change after intervention	−42%	(−60% to −18%)
CPE nonbacteremia clinical detection rate of CPE carriers ($\left(\delta_{c}\right)$		
Preintervention	0.0068 per day	(.0057–.0079)
Postintervention	0.0063 per day	(.0051–.0073)
Relative change after intervention	−7%	(−28% to +19%)

Abbreviations: CI, confidence interval; CPE, carbapenemase-producing Enterobacteriaceae.

The rate of progression to bacteremia, $\delta_{b}$, preintervention was 0.0018 per day (95% CI, .0015–.0021), postintervention $\delta_{b}$ was 0.0011 per day (95% CI, .0008–.0014), and relative change in $\delta_{b}$ after the intervention was −41% (95% CI, −60% to −18%). The rate of progression to nonbacteremia clinical detection, $\delta_{c}$, preintervention was 0.0068 per day (95% CI, .0057–.0079), postintervention $\delta_{c}$ was 0.0063 per day (95% CI, .0051–.0073), and the relative change in $\delta_{c}$ after the intervention was −7% (95% CI, −28% to +19%). These results were not sensitive to changes in assumed contact precaution effectiveness, clearance rate, or surveillance test sensitivity (Supplementary Figures 1 and 2).

## CONCLUSIONS

According to our model, contact precautions for surveillance-detected carriers alone could be responsible for the decrease in CPE carriage prevalence observed during the Chicago-area LTACH intervention [4]. If carriers under contact precautions transmitted CPE to other LTACH patients at a rate about 50% lower than carriers not under contact precautions, no additional changes to transmission rates were required to produce outcomes consistent with what was observed. In other words, if other components of the intervention, such as chlorhexidine bathing of all patients and healthcare worker hygiene monitoring, had a substantial effect on reducing transmissions, then the model suggests that the reduction in CPE carriage prevalence after the intervention should have been greater than observed.

This finding is sensitive to the assumption of 50% contact precaution effectiveness. The data do not provide direct support for the accuracy of this assumption, as they do not identify which patient acquisitions occurred via CPE organisms expelled by patients under contact precautions or by undetected carriers. If contact precautions had a smaller effect on reducing transmission, it is more likely that other components of the intervention bundle had a transmission-reducing effect. Contact precaution effectiveness would have to be less than about 20% (ie, detected carriers transmit at >80% the rate of undetected carriers) to convincingly conclude that other intervention components reduced transmission.

If it is true that our assumption for contact precaution effectiveness of ε=0.5 was substantially high, there are multiple mechanisms by which the intervention bundle could have caused a decrease in the baseline transmission rate β, that is, reduced transmission rates from any CPE carrier regardless of detection status. For example, the bundle included initiating daily bathing of all patients with cloths impregnated with 2% chlorhexidine gluconate, which could potentially prevent transmissions from any detected or undetected carrier via decreasing or protecting against skin contamination. The study estimated that enough cloths were delivered to the LTACHs for every patient to receive approximately one bath per day, and that training and assessment for bathing practices were conducted, but no other details about adherence or effectiveness were reported for this intervention component [4].

The intervention bundle also included “healthcare-worker education and adherence monitoring, with a focus on hand hygiene” [4]. A postintervention improvement in hand hygiene among healthcare workers could also have decreased the transmission rate from both detected and undetected carriers, contributing to a decrease in β. While the study reported observed rates of healthcare worker hand hygiene adherence during the intervention at room exit (70.8%) and room entry (24.4%), there were no preintervention adherence data for comparison [4].

While the above describe plausible explanations for the intervention results if contact precaution effects were much weaker than our 50% assumption, our findings also suggest that assuming highly effective contact precautions could also be consistent with the data. However, a high value of ε much greater than 50% is only possible if the baseline transmission rate β increased substantially after the intervention. A postintervention increase in β means that postintervention undetected carriers transmitted more per capita than preintervention undetected carriers, and postintervention detected carriers transmitted more per capita than preintervention clinically detected carriers. This scenario might be plausible if the increased volume of detected carriers requiring contact precautions resulted in an effort tradeoff where effectiveness decreased on a per-patient level among undetected patients and among detected patients. There is no evidence that this might have occurred in these LTACHs, and seems unlikely given the broad scope of the intervention components. However, this finding highlights the potential importance of further research into potential tradeoffs or unintended consequences of efforts focused on particular groups of patients.

Our findings strongly suggest a postintervention decrease in the per-capita rate of CPE carriers progressing to CPE bacteremia, that is, the decrease in the overall LTACH bacteremia incidence was not just due to the lower prevalence of CPE carriers at risk of infection, but also due to a decreased per-capita risk of infection among carriers. It is plausible that this was a result of chlorhexidine bathing or other hygiene-oriented components of the intervention, which could have reduced the risk of carried organisms entering the bloodstream.

Conversely, our findings do not conclusively suggest that the per-capita progression rate to nonbacteremia clinical detection changed after the intervention. While the overall incidence of CPE clinical detections decreased postintervention, the magnitude of decrease can be explained by the decrease in prevalence of carriers at risk of progression, and does not require a substantial change in the per-capita progression rate.

We did not model the possibility that the intervention caused an increase in the rate that CPE carriers cleared colonization. If intervention efforts did increase the clearance rate, then it is possible our model overestimated the intervention’s impact on transmission. There is evidence that chlorhexidine gluconate bathing might reduce skin colonization with CPE [16], but a recent review did not cite any studies about its effect on rectal colonization [17], and the surveillance testing that produced the target data for our model was done rectally.

If the intervention efforts reduced skin contamination on carriers but did not directly eliminate rectal carriage, then our finding that the intervention reduced the bacteremia progression rate but perhaps not the baseline transmission rate could have implications for inferring the sources of those events—that is, skin contamination may be an important source of organisms that initiate a bloodstream infection in that patient but may be less important as a source of organisms posing risk to other patients in the LTACH. Organisms carried rectally and in other internal sites could contaminate the hospital environment intermittently, for example, via stool or invasive devices, which could create sources of organisms that pose risk to other patients and would not be removed via skin decontamination. More research focused on these possibilities would be beneficial.

Insights derived from this work may or may not apply to other drug-resistant organisms. CPE are gram-negative bacteria that may have different contamination and transmission patterns in healthcare facilities than gram-positive bacterial strains such as methicillin-resistant *Staphylococcus aureus* or vancomycin-resistant *Enterococcus*, for which the effectiveness of contact precautions have been questioned [8]. However, the reasons why contact precautions might be more or less effective for preventing transmission of different types of bacteria are unclear; further research could shed light on potential organism-specific nuances relevant for transmission control.

Insights about the effect of contact precautions in LTACHs may not extend to other types of healthcare facilities. It is possible that atypical care required by LTACH patients could create unusual transmission pathways that lead to unusual effects of contact precautions. Also, long lengths of stay for LTACH patients (mean, >25 days) affects the way that transmission reduction manifests in observable outcomes. In short-stay hospitals, effects of prevented acquisitions may not be apparent if acquiring patients tend to be discharged before progressing to clinical infection, testing positive via surveillance, or transmitting to other hospital patients. Thus, the effects of reducing transmission in short-stay hospitals might be more difficult to observe without data from patients and their contacts at postdischarge locations.

Our model-based approach has several advantages. Its representation of patient states allows us to quantify the relationship between observable clinical events and unobservable colonization events, which makes it particularly well suited to examine the mechanisms of interventions that could affect both types of events simultaneously. Our model also flexibly accounts for length of stay and death rate and retains the realism that a patient’s discharge hazard over the course of a stay is nonconstant. The effects of those length of stay details on the relationship between facility prevalence, infection incidence, and nonlinear transmission processes are difficult to account for without a mathematical model.

Our approach to solving for key unknown model parameters by assuming an equilibrium state for pre- and postintervention models allowed us to make use of equation-based results that can be efficiently solved and do not require the use of extensive, time-consuming simulations. The equilibrium assumption is consistent with the observation that CPE prevalence was stable during the preintervention period and then restabilized a new level several months after the intervention efforts were in place [4]. Other scenarios in which unstable or transient levels of facility colonization are of interest would require modifications to our approach.

The use of a multifaceted “bundled” approach to intervening in healthcare facilities seeking to reduce the presence of drug-resistant organisms is understandable; the primary goal is to reduce risk to facility patients from dangerous infections, and if the projected effects of particular intervention components are uncertain then it makes sense to maximize the probability of success by combining them. For the secondary but still highly important goal of understanding the effect that each intervention component had on the outcomes, our work demonstrates that pairing mathematical transmission models with the data can be a powerful tool.

Our model-based findings also suggest additional data collection efforts that would help isolate the effects of contact precautions. Data that can suggest the source of patient acquisitions via genetic comparisons of patient samples would be particularly powerful. Combined with data on detection timing and contact precaution status, such genetic data could be used to quantify the relative rates of transmission from carriers under contact precautions vs other carriers, which would help constrain the possible scenarios depicted in Figure 2A. Genetic studies have been performed with samples from hospital patients with *Staphylococcus aureus* [18] and *Clostridioides difficile* [19]; we believe that insights derived from such work would be greatly enhanced by combining their data with epidemiological models designed to elucidate actionable insights for efficient transmission control.

## Supplementary Data

Supplementary materials are available at *Clinical Infectious Diseases* online. Consisting of data provided by the authors to benefit the reader, the posted materials are not copyedited and are the sole responsibility of the authors, so questions or comments should be addressed to the corresponding author.

ciz557_suppl_Supplementary_Information

## Notes

***Financial support.*** This work was supported by the Centers for Disease Control and Prevention (Prevention Epicenters award number U54CK000456 and MIND-Healthcare Program award number U01CK000538) and the Department of Veterans Affairs Health Services Research and Development Service (VA Center of Innovation award number I50HX001240).

***Potential conflicts of interest.*** All authors report no potential conflicts. All authors have submitted the ICMJE Form for Disclosure of Potential Conflicts of Interest. Conflicts that the editors consider relevant to the content of the manuscript have been disclosed.
