# Supplementary material for: Model-based Assessment of the Effect of Contact Precautions Applied to Surveillance-detected Carriers of Carbapenemase-producing Enterobacteriaceae in Long-term Acute Care Hospitals
Source: Clin Infect Dis. 2019 Sep 13;69(Suppl 3):S206–13. doi: 10.1093/cid/ciz557 (PMC6761367; doi:10.1093/cid/ciz557)
Supplement: ciz557_suppl_Supplementary_Information [file ciz557_suppl_supplementary_information.pdf]

Supplementary methods

Death rate and length of stay and settings

We assume a constant death rate ω (per day) for all patients, and that the length of stay of live-discharge patients is governed by a distribution with cumulative distribution function $F(t)$ and probability density function $f(t)$. We are given the fraction of stays ending in death, p_d:

$$p_d = \int_0^\infty \omega e^{-\omega t}\big(1 - F(t)\big)dt$$

Using integration by parts we can rewrite this as

$$p_d = 1 - \int_0^\infty e^{-\omega t} f(t)dt = 1 - M(-\omega)$$

Where M is the moment-generating function of the live-discharge length of stay distribution.

We are also given the mean and 25th, 50th, and 75th percentiles of the overall length of stay distribution (μ, l_{25}, l_{50}, l_{75}), which encompasses stays ending in either death or live discharge. Under the above assumptions we have the following cumulative distribution function F_{all} for overall length of stay:

$$F_{\text{all}}(t) = \int_0^t \Big(\omega e^{-\omega\tau}\big(1 - F(\tau)\big) + e^{-\omega\tau} f(\tau)\Big)\, d\tau$$

Using integration by parts we get

$$F_{\text{all}}(t) = 1 - e^{-\omega t}\big(1 - F(t)\big)$$

The mean is therefore:

$$\mu = \int_0^\infty \big(1 - F_{\text{all}}(t)\big)dt = \int_0^\infty e^{-\omega t}\big(1 - F(t)\big)dt = \frac{1 - \int_0^\infty e^{-\omega t} f(t)dt}{\omega} = \frac{1 - M(-\omega)}{\omega}$$

Noting the result above that $p_d = 1 - M(-\omega)$, we can solve for ω independently of the live-discharge stay distribution:

$$\omega = \frac{p_d}{\mu}$$

Then we have four equations constraining the live-discharge stay distribution:

$$\mu = \frac{1 - M(-\omega)}{\omega}$$

$$0.25 = 1 - e^{-\omega l_{25}}\big(1 - F(l_{25})\big)$$

$$0.5 = 1 - e^{-\omega l_{50}}\big(1 - F(l_{50})\big)$$

$$0.75 = 1 - e^{-\omega l_{75}}\big(1 - F(l_{75})\big)$$

We assume a mixed exponential-gamma distribution for the live-discharge stay distribution, with a portion p_x of patients following an exponential distribution with mean μ_x and the rest following a gamma distribution with mean μ_g and shape parameter k. For this distribution:

$$M(x) = p_x(1 - \mu_x x)^{-1} + (1 - p_x)\big(1 - \mu_g x/k\big)^{-k}$$

$$F(t) = p_x\left(1 - e^{-t/\mu_x}\right) + (1 - p_x)\frac{1}{\Gamma(k)}\int_0^{kt/\mu_g} \tau^{k-1}e^{-\tau}d\tau$$

We apply these two functions to the four equations above and numerically solve for the four unknown parameters p_x, μ_x, μ_g, and k.

Pre-intervention, we have $p_d = 0.215$, $\mu = 33.8$ days, $l_{25} = 16$ days, $l_{50} = 28$ days, and $l_{75} = 43$ days, which leads to:

$$\omega = 0.006361503,\ p_x = 0.4626888,\ \mu_x = 49.8726425,\ \mu_g = 34.3445429, \text{ and } k = 5.3106238$$

Post-intervention, we have $p_d = 0.176$, $\mu = 30.5$ days, $l_{25} = 16$ days, $l_{50} = 26$ days, and $l_{75} = 39$ days, which leads to:

$$\omega = 0.005767406,\ p_x = 0.1800357,\ \mu_x = 53.7929745,\ \mu_g = 31.5245810, \text{ and } k = 3.7254548$$

Patient state dynamics

Given a system

$$\frac{d\mathbf{x}}{dt} = \mathbf{W}t, \qquad \mathbf{x}(0) = \mathbf{x}_\mathrm{a}$$

Where $\mathbf{W}$ governs during-stay state transitions and death rates, $\mathbf{x}_\mathrm{a}$ is the distribution of states at admission, and the live-discharge length of stay distribution has cumulative distribution function $F(t)$, the equilibrium cross-sectional state distribution $\mathbf{x}^*$ is:

$$\mathbf{x}^* = \frac{\left(\int_0^\infty e^{\mathbf{W}t}\left(1 - F(t)\right)dt\right)\mathbf{x}_\mathrm{a}}{\mathbf{1}^\mathrm{T}\left(\int_0^\infty e^{\mathbf{W}t}\left(1 - F(t)\right)dt\right)\mathbf{x}_\mathrm{a}}$$

To evaluate the integral in this expression, we must consider the integral $\int_0^\infty \varphi_i(t)\left(1 - F(t)\right)dt$ for the eigenfunctions φ that comprise the elements of the matrix exponential $e^{\mathbf{W}t}$. Assuming the death hazard for any patient is nonzero, the eigenvalues λ_i of $\mathbf{W}$ are negative and real, so the eigenfunctions take the form $\varphi_i(t) = e^{\lambda_i t}$. We use integration by parts:

$$\int_0^\infty e^{\lambda_i t}\left(1 - F(t)\right)dt = \frac{1}{\lambda_i}\left[\left(1 - F(t)\right)e^{\lambda_i t}\right]_0^\infty + \frac{1}{\lambda_i}\int_0^\infty e^{\lambda_i t}f(t)dt = \frac{1}{\lambda_i}(0 - 1) + \frac{1}{\lambda_i}M(\lambda_i) = \frac{M(\lambda_i) - 1}{\lambda_i}$$

Here, f and M are the probability density function and moment-generating function of the live-discharge length of stay distribution, respectively.

Pre-intervention dynamics:

$$\frac{dS}{dt} = -(\alpha + \omega)S + \gamma C, \qquad S(0) = 1 - p_\mathrm{a}$$

$$\frac{dC}{dt} = \alpha S - (\delta_\mathrm{c} + \delta_\mathrm{b} + \gamma + \omega)C, \qquad C(0) = p_\mathrm{a}$$

$$\frac{dC_\mathrm{cd}}{dt} = \delta_\mathrm{c}C - (\delta_\mathrm{b} + \omega)C_\mathrm{cd}, \qquad C_\mathrm{cd}(0) = 0$$

$$\frac{dC_\mathrm{b}}{dt} = \delta_\mathrm{b}C + \delta_\mathrm{b}C_\mathrm{cd} - \omega C_\mathrm{b}, \qquad C_\mathrm{b}(0) = 0$$

$$\mathbf{W}_{\text{pre}} = \begin{pmatrix} -\alpha-\omega & \gamma & 0 & 0 \\ \alpha & -\delta_\text{c}-\delta_\text{b}-\gamma-\omega & 0 & 0 \\ 0 & \delta_\text{c} & -\delta_\text{b}-\omega & 0 \\ 0 & \delta_\text{b} & \delta_\text{b} & -\omega \end{pmatrix}, \qquad \mathbf{x}_\text{a} = \begin{pmatrix} 1-p_\text{a} \\ p_\text{a} \\ 0 \\ 0 \end{pmatrix}$$

$$\alpha = \beta\big(C^* + (1-\varepsilon)(C^*_\text{cd} + C^*_\text{b})\big)$$

Our constraints are

$$C^* + C^*_\text{cd} + C^*_\text{b} = p_\text{pre}$$

$$(\delta_\text{c} + \delta_\text{b})C^* = d_\text{pre}$$

$$\delta_\text{b}(C^* + C^*_\text{cd}) = b_\text{pre}$$

In the above equations, the values of p_a, p, d, and b are fixed from the pre-intervention results from the reported data (Table 1 main text), with p_a and p scaled by an assumed surveillance test sensitivity (Table 2 main text). The values of γ and ε are assumed (Table 2 main text). The value of the death rate ω and the length of stay distribution formula *F* are fixed at the pre-intervention values described above. Then we solve for α, δ_c, δ_b, and the equilibrium $\mathbf{x}^* = (S^*, C^*, C^*_\text{cd}, C^*_\text{b})$ by simultaneously solving the above equation $\mathbf{x}^*$ with $\mathbf{W} = \mathbf{W}_\text{pre}$ and the three above constraint equations. Finally, we solve for β using the remaining equation above for α.

Post-intervention dynamics:

$$\frac{dS}{dt} = -(\alpha+\omega)S + \gamma C, \qquad S(0) = 1 - p_\text{a}$$

$$\frac{dS_\text{sd}}{dt} = -\big((1-\varepsilon)\alpha + \omega\big)S_\text{sd} + \gamma C_\text{sd}, \qquad S_\text{sd}(0) = 0$$

$$\frac{dC}{dt} = \alpha S - (\delta_\text{s} + \delta_\text{c} + \delta_\text{b} + \gamma + \omega)C, \qquad C(0) = (1-\pi_\text{a})p_\text{a}$$

$$\frac{dC_\text{sd}}{dt} = \varepsilon\alpha S_\text{sd} + \delta_\text{s}C - (\delta_\text{c} + \delta_\text{b} + \gamma + \omega)C_\text{sd}, \qquad C(0) = \pi_\text{a}p_\text{a}$$

$$\frac{dC_\text{cd}}{dt} = \delta_\text{c}C + \delta_\text{c}C_\text{sd} - (\delta_\text{b} + \omega)C_\text{cd}, \qquad C_\text{cd}(0) = 0$$

$$\frac{dC_\text{b}}{dt} = \delta_\text{b}C + \delta_\text{b}C_\text{sd} + \delta_\text{b}C_\text{cd} - \omega C_\text{b}, \qquad C_\text{b}(0) = 0$$

$$\mathbf{W}_\text{post} = \begin{pmatrix} -\alpha-\omega & 0 & \gamma & 0 & 0 & 0 \\ 0 & -(1-\varepsilon)\alpha-\omega & 0 & \gamma & 0 & 0 \\ \alpha & 0 & -\delta_\text{s}-\delta_\text{c}-\delta_\text{b}-\gamma-\omega & 0 & 0 & 0 \\ 0 & \varepsilon\alpha & \delta_\text{s} & -\delta_\text{c}-\delta_\text{b}-\gamma-\omega & 0 & 0 \\ 0 & 0 & \delta_\text{c} & \delta_\text{c} & -\delta_\text{b}-\omega & 0 \\ 0 & 0 & \delta_\text{b} & \delta_\text{b} & \delta_\text{b} & -\omega \end{pmatrix},$$

$$\mathbf{x}_\text{a} = \begin{pmatrix} 1-p_\text{a} \\ 0 \\ (1-\pi_\text{a})p_\text{a} \\ \pi_\text{a}p_\text{a} \\ 0 \\ 0 \end{pmatrix}$$

$$\alpha = \beta\left(C^* + (1-\varepsilon)(C_{\mathrm{sd}}^* + C_{\mathrm{cd}}^* + C_{\mathrm{b}}^*)\right)$$

Our constraints are

$$C^* + C_{\mathrm{sd}}^* + C_{\mathrm{cd}}^* + C_{\mathrm{b}}^* = p_{\mathrm{post}}$$

$$(\delta_{\mathrm{c}} + \delta_{\mathrm{b}})(C^* + C_{\mathrm{sd}}^*) = d_{\mathrm{post}}$$

$$\delta_{\mathrm{b}}(C^* + C_{\mathrm{sd}}^* + C_{\mathrm{cd}}^*) = b_{\mathrm{post}}$$

In the above equations, the values of p_{a}, p, d, b, π_{a}, and δ_{s} are fixed from the post-intervention results from the reported data (Table 1 main text), with p_{a}, p, π_{a}, and δ_{s} scaled by an assumed surveillance test sensitivity (Table 2 main text). The values of γ and ε are assumed (Table 2 main text). The value of the death rate ω and the length of stay distribution formula F are fixed at the post-intervention values described above. Then we solve for α, δ_{c}, δ_{b}, and the equilibrium $\mathbf{x}^* = (S^*, S_{\mathrm{sd}}^*, C^*, C_{\mathrm{sd}}^*, C_{\mathrm{cd}}^*, C_{\mathrm{b}}^*)$ by simultaneously solving the above equation $\mathbf{x}^*$ with $\mathbf{W} = \mathbf{W}_{\mathrm{post}}$ and the three above constraint equations. Finally, we solve for β using the remaining equation above for α.

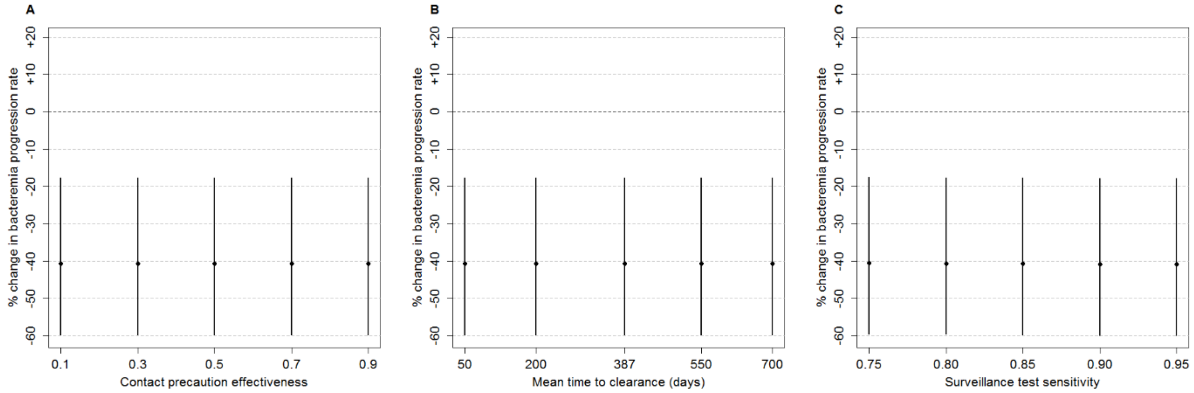

Supplementary Figure 1 - Effect of assumptions on bacteremia progression rate results.

Vertical axis is the % change from pre- to post-intervention of the result for δ_{b}, the per-capita progression rate to bacteremia for CPE carriers. Panels show the sensitivity of this result to changes in single parameters from the default (default is the middle value in each panel). Circles: mean results; vertical lines: 95% confidence intervals. Panel A: effectiveness of contact precautions is ε, so the per-capita acquisition rate of non-isolated susceptible patients is $\beta_{\mathrm{pre}}\big(C+(1-\varepsilon)(C_{\mathrm{cd}}+C_{\mathrm{b}})\big)$ pre-intervention and $\beta_{\mathrm{post}}\big(C+(1-\varepsilon)(C_{\mathrm{sd}}+C_{\mathrm{cd}}+C_{\mathrm{b}})\big)$ post-intervention. Panel B: mean time to clearance is $1/\gamma$ days, where γ is the rate at which non-clinically infected CPE carriers clear colonization and become susceptible to re-acquisition. Panel C: surveillance test sensitivity is 1 minus the probability that CPE carriers falsely test negative at surveillance.

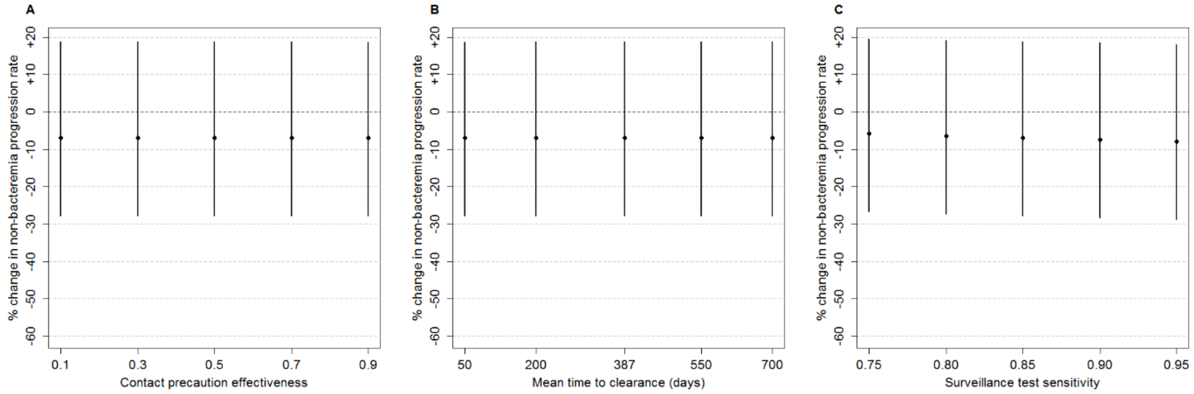

Supplementary Figure 2 – Effect of assumptions on non-bacteremia clinical detection rate results.

Vertical axis is the % change from pre- to post-intervention of the result for δ_c, the per-capita progression rate to non-bacteremia clinical detection for CPE carriers. Panels show the sensitivity of this result to changes in single parameters from the default (default is the middle value in each panel). Circles: mean results; vertical lines: 95% confidence intervals. Panel A: effectiveness of contact precautions is ε, so the per-capita acquisition rate of non-isolated susceptible patients is $\beta_{\text{pre}}\left(C + (1-\varepsilon)(C_{\text{cd}} + C_{\text{b}})\right)$ pre-intervention and $\beta_{\text{post}}\left(C + (1-\varepsilon)(C_{\text{sd}} + C_{\text{cd}} + C_{\text{b}})\right)$ post-intervention. Panel B: mean time to clearance is $1/\gamma$ days, where γ is the rate at which non-clinically infected CPE carriers clear colonization and become susceptible to re-acquisition. Panel C: surveillance test sensitivity is 1 minus the probability that CPE carriers falsely test negative at surveillance.
